# Anaphylactic reaction caused by skin contact with the disinfectant chloramine‐T

**DOI:** 10.1111/cod.13200

**Published:** 2019-02-19

**Authors:** Berrit M. Roorda, Hans L.A. Nienhuis, Marie L.A. Schuttelaar

**Affiliations:** ^1^ Department of Dermatology, University of Groningen University Medical Centre Groningen Groningen The Netherlands; ^2^ Department of Internal Medicine, University of Groningen University Medical Centre Groningen Groningen The Netherlands

**Keywords:** anaphylaxis, CAS no. 7080‐50‐4, case report, chloramine‐T, contact urticaria, disinfectant, Halamid

Chloramine‐T (CAS no. 7080‐50‐4, syn. sodium *p*‐toluenesulfonchloramide) is a crystalline powder with a chlorine basis, and is commonly used as a sterilizer, antiseptic, disinfectant, and chemical reagent. Sensitization is often work‐related. We report a case of an anaphylactic reaction to chloramine‐T.

## CASE REPORT

**Figure 1:**
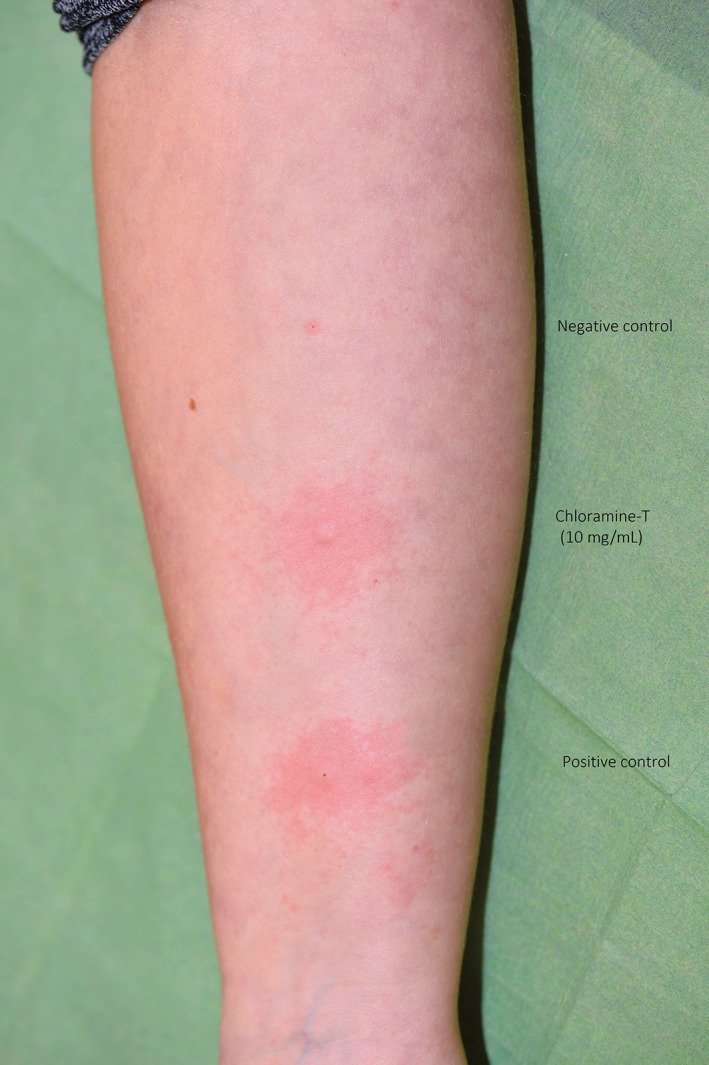
Positive prick test (wheal and flare) reaction to chloramine‐T after 15 minutes

A 32‐year‐old healthy non‐atopic female with no history of asthma showed generalized itchy erythema, dyspnoea and vertigo 15 minutes after cooling a second‐degree burn on her left underarm in water with added chloramine‐T (Halamid). She was diagnosed with anaphylaxis, and observed and treated with 0.5 mg of intramuscular adrenaline and 2 mg of intravenous clemastine at the hospital. Some hours later, she was discharged, with only diffuse mild erythema remaining. She had performed cleaning activities at a butchery for 17 years without using gloves. She had regularly developed localized wheals after skin contact with chloramine‐T. We performed prick tests with an in‐house preparation of 10 mg/mL of the patient's product. Readings were performed after 15 minutes. Physiological salt as a negative control caused no wheal or flare. Histamine, as a positive control, and chloramine‐T caused erythematous wheals and flares with mean diameters of 6 and 12.5 mm, respectively (Figure 1). Prick tests performed in three controls gave negative results. Laboratory tests showed a chloramine‐T‐specific IgE level of >100 kUA/L (values >0.34 kU/L were defined as positive) and a total IgE level of 870 kU/L (normal: 0‐115 kU/L) (ImmunoCAP; ThermoFisher Scientific, Uppsala, Sweden).

## DISCUSSION

This is the first report of an anaphylactic reaction caused by immediate‐type hypersensitivity to chloramine‐T. On review of the literature, we found several case reports describing urticaria, rhinitis and asthmatic bronchial obstruction caused by chloramine‐T after skin contact or airborne exposure.^1^, ^2^, ^3^, ^4^, ^5^, ^6^, ^7^, ^8^ Dooms‐Goossens et al described a nurse with contact urticaria, dyspnoea and rhinitis after skin contact and airborne exposure to chloramine‐T powder.^5^ Kujala et al reported a bath attendant with rhinitis and asthma after spraying the workplace with a chloramine‐T solution.^6^ Kanerva et al described a hospital bath attendant with contact urticaria and rhinitis after disinfecting surfaces in hospital bath rooms with chloramine‐T solution.^7^ Our patient was probably sensitized to chloramine‐T during her cleaning activities in the last 17 years, producing chloramine‐T‐specific IgE antibodies. After binding of chloramine‐T IgE antibodies to the mast cells and basophils, they become more sensitive for degranulation. When re‐exposure to chloramine‐T occurs, they degranulate (sooner). When chloramine‐T binds to the IgE‐loaded mast cells, it triggers the release of vasoactive substances such as histamine and tryptase. It is likely that our patient was exposed to a relatively large amount of chloramine‐T through the burn wound, and that this triggered massive degranulation of mast cells, resulting in an anaphylactic reaction. After replacement of chloramine‐T with chlorine at the workplace, the patient was free of symptoms.

## CONFLICTS OF INTEREST

The authors have no conflicts of interest to report.
